# Irradiation-Modulated Murine Brain Microenvironment Enhances GL261-Tumor Growth and Inhibits Anti-PD-L1 Immunotherapy

**DOI:** 10.3389/fonc.2021.693146

**Published:** 2021-06-24

**Authors:** Joel R. Garbow, Tanner M. Johanns, Xia Ge, John A. Engelbach, Liya Yuan, Sonika Dahiya, Christina I. Tsien, Feng Gao, Keith M. Rich, Joseph J. H. Ackerman

**Affiliations:** ^1^ Department of Radiology, Washington University, Saint Louis, MO, United States; ^2^ Alvin J. Siteman Cancer Center, Washington University, Saint Louis, MO, United States; ^3^ Department of Internal Medicine, Washington University, Saint Louis, MO, United States; ^4^ Department of Neurosurgery, Washington University, Saint Louis, MO, United States; ^5^ Division of Neuropathology, Department of Pathology and Immunology, Washington University, Saint Louis, MO, United States; ^6^ Department of Radiation Oncology, Washington University, Saint Louis, MO, United States; ^7^ Department of Surgery, Washington University, Saint Louis, MO, United States; ^8^ Department of Chemistry, Washington University, Saint Louis, MO, United States

**Keywords:** MRI, radiation, tumor, microenvironment, immunotherapy, checkpoint inhibitors, microglia

## Abstract

**Purpose:**

Clinical evidence suggests radiation induces changes in the brain microenvironment that affect subsequent response to treatment. This study investigates the effect of previous radiation, delivered six weeks prior to orthotopic tumor implantation, on subsequent tumor growth and therapeutic response to anti-PD-L1 therapy in an intracranial mouse model, termed the Radiation Induced Immunosuppressive Microenvironment (RI^2^M) model.

**Method and Materials:**

C57Bl/6 mice received focal (hemispheric) single-fraction, 30-Gy radiation using the Leksell GammaKnife^®^ Perfexion™, a dose that does not produce frank/gross radiation necrosis. Non-irradiated GL261 glioblastoma tumor cells were implanted six weeks later into the irradiated hemisphere. Lesion volume was measured longitudinally by *in vivo* MRI. In a separate experiment, tumors were implanted into either previously irradiated (30 Gy) or non-irradiated mouse brain, mice were treated with anti-PD-L1 antibody, and Kaplan-Meier survival curves were constructed. Mouse brains were assessed by conventional hematoxylin and eosin (H&E) staining, IBA-1 staining, which detects activated microglia and macrophages, and fluorescence-activated cell sorting (FACS) analysis.

**Results:**

Tumors in previously irradiated brain display aggressive, invasive growth, characterized by viable tumor and large regions of hemorrhage and necrosis. Mice challenged intracranially with GL261 six weeks after prior intracranial irradiation are unresponsive to anti-PD-L1 therapy. K-M curves demonstrate a statistically significant difference in survival for tumor-bearing mice treated with anti-PD-L1 antibody between RI^2^M *vs.* non-irradiated mice. The most prominent immunologic change in the post-irradiated brain parenchyma is an increased frequency of activated microglia.

**Conclusions:**

The RI^2^M model focuses on the persisting (weeks-to-months) impact of radiation applied to normal, control-state brain on the growth characteristics and immunotherapy response of subsequently implanted tumor. GL261 tumors growing in the RI^2^M grew markedly more aggressively, with tumor cells admixed with regions of hemorrhage and necrosis, and showed a dramatic loss of response to anti-PD-L1 therapy compared to tumors in non-irradiated brain. IHC and FACS analyses demonstrate increased frequency of activated microglia, which correlates with loss of sensitivity to checkpoint immunotherapy. Given that standard-of-care for primary brain tumor following resection includes concurrent radiation and chemotherapy, these striking observations strongly motivate detailed assessment of the *late effects* of the RI^2^M on tumor growth and therapeutic efficacy.

## Introduction

Radiotherapy combined with immunotherapy is an active area of investigation in the treatment of brain tumors. Active areas of study include investigations of (i) radiation dose and fractionation required to induce immunologic cell death (1); (ii) concurrent *vs*. sequential therapies (2–4); and (iii) outcomes following whole brain radiation therapy *vs*. stereotactic radiosurgery.

Clinical evidence suggests radiation induces changes in the brain microenvironment that affect subsequent response to treatment. Studies of patients with metastatic brain tumors noted metastatic lesions that progress after initial irradiation are often less responsive to subsequent treatment. Previously irradiated melanoma and NSCLC brain metastases failed to respond to pembrolizumab, while non-irradiated lesions had similar response rate to those of extracranial disease (5). Similar observations were noted in a separate cohort of patients with melanoma brain metastases, in which the cohort with lesions that progressed following prior irradiation had a substantially lower response rate to immunotherapy compared to the cohort with irradiation-naïve lesions (6). In recurrent glioblastoma, PD-1 monotherapy and PD-1/CTLA-4 combination therapy alone failed to demonstrate clinical benefit or objective response rates (7–9). These patients were all treated previously with chemoradiotherapy per standard-of-care. Together, these clinical observations suggest that late effects of prior irradiation to the brain microenvironment may be associated with resistance to immune checkpoint inhibition (ICI) therapy.

We have developed a novel, Gamma Knife^®^ (GK) enabled, focal (hemispheric) brain-irradiation mouse model (10), termed the Radiation-Induced Immunosuppressive Microenvironment (RI^2^M) model, that provides a powerful platform for investigation into the late effects of irradiation on the brain parenchyma microenvironment. Earlier studies of GK-enabled focal-irradiation of mouse brain from this laboratory employed substantially greater radiation doses and were purposefully designed to reliably elicit late-time-to-onset radiation necrosis in an experimentally tractable time frame, with radiation necrosis consistently appearing approximately four to eight weeks post-irradiation (11–17). Importantly, the resultant radiation necrosis in the mouse brain recapitulated all of the key histologic hallmarks of the clinically observed pathology, giving strong confidence regarding the platform’s clinical relevance.

In a recent study (10), we observed that non-irradiated DBT glioblastoma cells, implanted into the RI^2^M of syngeneic mice six weeks post-irradiation – thus, absent acute radiation effects – showed remarkable changes in growth characteristics. Specifically, tumors displayed aggressive, invasive growth, characterized by viable tumor and large regions of hemorrhage and necrosis, resulting in decreased survival compared to tumors growing in non-irradiated brain. Importantly, for these studies, we employed irradiation doses that elicit no frank/gross evidence (MRI and H&E histology) of radiation necrosis, or other outward pathology (e.g., behavior), in the post-irradiation setting. Thus, these data suggest that orthotopic tumors originating from naïve (non-irradiated) glioblastoma cells growing in the previously (six weeks) irradiated brain show many of the histologic hallmarks of recurrent GBM in patients.

Utilizing this model, we are able to evaluate the effects of prior irradiation of the brain parenchyma on the efficacy of a known immune checkpoint sensitive orthotopic transplant glioblastoma model, GL261. We demonstrate that mice challenged intracranially with non-irradiated (naïve) GL261 cells after prior (six weeks) intracranial irradiation with a dose eliciting no frank/gross evidence of radiation necrosis or other pathology are unresponsive to anti-PD-1/PD-L1 directed therapy. The observations of enhanced tumor growth and resistance to checkpoint inhibitors for tumors growing in RI^2^M are distinct from studies employing combined radiation and immunotherapy to treat existing tumors (18, 19). The most prominent immunologic change in the post-irradiated brain parenchyma is an increased frequency of activated microglia, suggesting they may play a role in the immunosuppressive effects observed. These striking findings have important implications regarding the clinical effects of prior, standard-of-care irradiation on the brain parenchyma and on the subsequent use of ICI therapy in patients with brain tumors. Specifically, a better understanding of the delayed effects of irradiation on the brain/tumor microenvironment will be crucial to identifying effective therapies that can safely synergize with immune checkpoint inhibitors to enhance immune responsiveness and improve outcome for brain tumor patients.

## Materials and Methods

### Animals

All experiments were performed in accordance with the guidelines of Washington University’s Institutional Animal Care and Use Committee and were approved by that committee. Seven-to eight-week-old female C57Bl/6 mice (Envigo Laboratories, Indianapolis, IN), housed five per cage in a light- and temperature-controlled facility, were used in this study. These mice were observed daily to ensure that interventions were well tolerated. A subset of healthy-appearing mice was sacrificed for histology, and mice were also euthanized if they lost more than 20% body weight or suffered obvious behavioral deficits (e.g., ataxia).

### Gamma Knife Irradiation

Mice were anesthetized and restrained on a custom-built platform mounted to the stereotactic frame that attaches to the treatment couch of the Leksell GK Perfexion™ (Elekta, Stockholm, Sweden), a device used for stereotactic radiosurgery of patients with malignant brain tumors. Mice were anesthetized with a mixture of ketamine (25 mg/kg) and xylazine (5 mg/kg), injected intraperitoneally (IP) five minutes before the start of irradiation. Single 30-Gy radiation fractions (50% isodose), generated using the GK’s 4-mm collimator, were focused on the left cortex at a site ~ 3 mm posterior to bregma.

### Tumor Implantation

Tumor cells were implanted in mice, as described previously (20). Briefly, mice were anesthetized with isoflurane and secured in a stereotactic head holder. Murine GL261 glioblastoma cells were implanted (~50,000 cells suspended in 10 μL per mouse) over three minutes in the striatum at a site 2-mm posterior and 3-mm to the left of bregma, 2-mm below the cortical surface.

### Experimental Scheme

These experiments were designed to assess tumor growth and response to anti-PD-L1 immunotherapy in the setting of previously irradiated brain tissue. Cohorts of mice received a single fraction dose of 0 or 30 Gy (50% isodose), respectively, of GK radiation. At a radiation dose of 30 Gy, no frank radiation necrosis is observed, visualized by either anatomic MR imaging or standard H&E staining, up to 20 weeks post irradiation (19). Naïve (non-irradiated) GL261 tumor cells were implanted into the ipsilateral hemisphere six weeks post-brain-irradiation – thus obviating acute radiation effects – to evaluate the consequences for tumor growth and immuno-therapeutic response imposed by the RI^2^M. Mice treated with anti-PD-L1 antibody received IP injections on days 3, 6, 9, 12, and 15 post tumor implantation; untreated mice received injections of PBS vehicle on these same days (21).

### Magnetic Resonance Imaging

Imaging was performed with a 4.7-T small-animal MR scanner (Agilent/Varian, Santa Clara, CA) employing an actively decoupled coil pair: a 9-cm inner diameter volume coil (transmit) and a 1.5-cm outer diameter surface coil (receive). Before all imaging experiments, mice were anesthetized with isoflurane/O_2_ [2% (vol/vol)] and maintained on isoflurane/O_2_ [1% (vol/vol)] throughout the experiment. Mice were restrained in a laboratory-built, three-point, Teflon head holder and were placed on a water pad with circulating warm water to maintain body temperature at approximately 37 ± 1°C. Before being placed into the magnet, each mouse was injected intraperitoneally with 0.25 mL of MultiHance (gadobenate dimeglumine; Bracco Diagnostics Inc, Princeton, NJ) contrast agent, diluted 2:10 in sterile saline. This procedure highlights regions of impaired blood brain barrier integrity *via* vascular leakage of contrast agent into the parenchyma.

Mice were imaged on post-implantation (GL261 cells) days 10, 14, and 18, and then, every two-to-three weeks, until they were sacrificed, or died due to disease progression. Post-contrast T1-weighted images were acquired with the following parameters: time-to-repetition (TR) = 650 ms, time-to-echo (TE) = 11 ms, number of transient (NT) = 4, field of view = 15 x 15 mm^2^, matrix size = 128 x 128, slice thickness = 0.5 mm, 21 slices to cover the whole brain. T2-weighted images were collected with time-to-repetition (TR) = 1200 ms and time-to-echo (TE) = 50 ms, with all other parameters the same as for the T1W images.

### Histology

Mice were sacrificed and their brains were immediately removed from the skulls and immersed in formalin. After 24 hours, brains were transferred to a 20% alcohol solution. A 3-mm thick transaxial block, centered at the irradiation site (~3 mm behind the bregma), was obtained from each brain. The blocks were then processed through graded alcohols and embedded in paraffin. All paraffin-fixed blocks were sectioned from the center, at a thickness of five microns. Tissue sections were stained with hematoxylin and eosin (H&E) according to standard protocols.

To measure levels of activated microglia, 5-micron thick tissue sections were immunostained using a rabbit monoclonal anti-IBA-1 antibody (1:1000; Abcam, Cambridge, MA USA), followed by incubation with SuperPicture Polymer Detection Kit, HRP (Life Technologies, Frederick, MD, USA). Slides were viewed with a Hamamatsu NanoZoomer 2.0-HT whole slide imaging system (Hamamatsu Photonics, Bridgewater Township, NJ USA). All histologic and immunohistochemical analyses were performed by a board-certified neuropathologist (S.D.).

### Isolation of Tumor-Infiltrating Lymphocytes and Flow Cytometry Analysis

Flow cytometry experiments were performed on a separate cohort of animals that was not included in the survival study. Mice were sacrificed at post-implantation day 14 and intracranial tumors were harvested. Tumor-infiltrating leukocytes (TIL) were isolated by generating a single cell suspension through mechanical dissociation of the tumor tissue. Myelin was removed using a 30% Percoll density gradient. Red blood cells were removed using ACK lysis buffer. The resulting cell pellet was stained with fluorophore-conjugated antibodies to CD45, CD3, CD4, CD8, NK1.1, CD11b, Gr-1, and Zombie NIR (live/dead). All antibodies were obtained through BioLegend (San Diego, CA). Flow cytometry was performed on a BD LSRFortessa flow cytometer (BD Biosciences, San Jose, CA). Analysis was performed through FlowJo software (BD Biosciences). Statistical analysis was performed using the Student t-test in Prism (GraphPad Software, San Diego, CA).

Live CD45^+^ TIL were subgated into lymphoid and myeloid subsets to determine relative frequency among total TIL. Lymphoid cell populations were defined as CD4 or CD8 T cells (CD3^+^ NK1.1^-^ CD11b), NK cells (CD3^-^ NK1.1^+^ CD11b^-^), or NKT cells (CD3^+^ NK1.1^+^ CD11b^-^). Myeloid cell populations were defined as CD3^-^ NK1.1^-^ CD11b^+^, and further gated on granulocytic MDSC (Gr-MDSC; CD11b^lo^ Gr-1^hi^), monocytic MDSC (M-MDSC; CD11b^hi^ Gr-1^lo^), or tumor-associated macrophage/microglia (CD11b^+^ Gr-1^-^). Gating on resting microglia (CD45^lo^), activated microglia (CD45^int^), and tumor-associated macrophage (CD45^hi^) was performed on the CD11b^+^ Gr-1^-^ subset.

### Antibody Treatment

Monoclonal mouse anti-PD-L1 antibody (InVivoMAb, clone 10F.92G) was purchased from Bio X Cell (West Lebanon, NH) and diluted in Bio X Cell InVivoPure dilution buffer prior to use. 500 µg of antibody per mouse was injected IP on days 3, 6, 9, 12, and 15 post-tumor-implantation.

### Data Analysis and Statistics

Tumor volumes were derived using ImageJ (https://imagej.nih.gov/ij/), with regions of interest (ROIs) for the tumor lesions being drawn manually on the post-contrast T1-weighted images. In calculating lesion volumes, hypointense regions within the tumor were also treated as part of the lesions. MR-derived lesion volumes were calculated as the sum of the number of lesion voxels multiplied by the voxel volume. Changes in lesion volume over time were described using a linear mixed model, to account for potential correlation among multiple measurements from the same mouse, followed by *ad hoc* comparisons for between-group differences at each time point. Square-root transformation of lesions volumes was performed to better satisfy the normality and homoscedasticity assumptions for the linear mixed model. Distributions of survival times were described using the Kaplan-Meier (K-M) product limit method, and between-group differences were compared using a weighted log-rank test (22). All tests were two-sided and significance was set at a p-value of 0.05. Statistical analyses were performed using SAS 9.4 (SAS Institutes; Cary, NC).

## Results

### Tumor Implanted Into Previously Irradiated Brain Grows More Aggressively and Hemorrhagically

**Figure 1:**
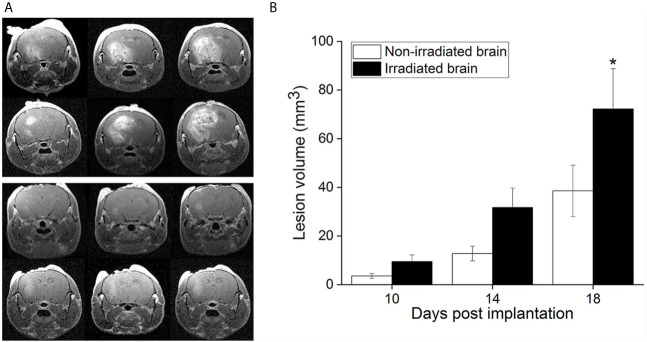
**(A)** Representative, contrast-enhanced T1-weighted MR images of GL261 tumor-bearing C57Bl/6 mice, collected 18 days after tumor implantation. The top panel shows three contiguous image slices for two animals whose brains were focally irradiated with 30-Gy (50% isodose) Gamma Knife radiation six weeks prior to GL261 cell implantation in the RI^2^M. The bottom panel shows similar images for non-irradiated mice. Tumors growing in the RI^2^M are larger and more hyperintense (reflecting greater leakage of contrast agent) compared with tumors growing in non-irradiated brain. **(B)** Plots showing mean lesion volume (+/- SEM) for tumors growing in irradiated (n = 9; black) and non-irradiated (n = 10; white) brain at post-implantation days 10, 14, and 18. At post-implantation day 14, there is a trend toward larger lesion volumes in irradiated brain (p = 0.07). At post-implantation day 18, the difference in lesion volumes is statistically significant (*p = 0.030).

**Figure 2:**
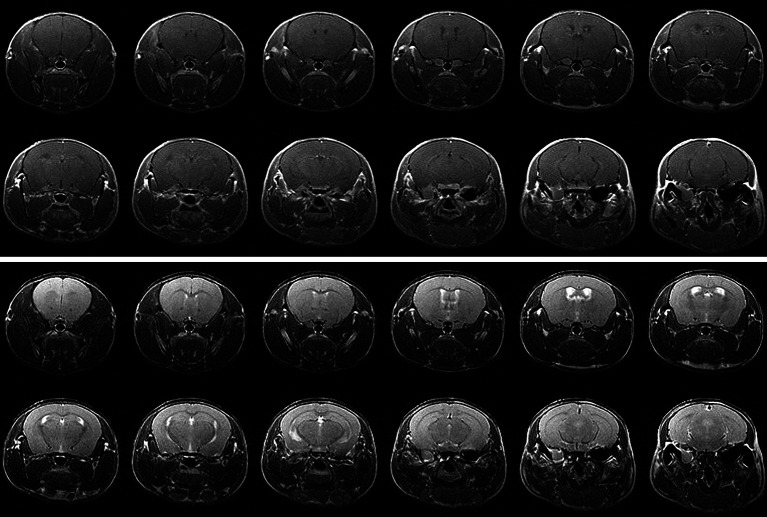
(top) Contrast-enhanced T1-weighted and (bottom) T2-weighted MR images of a C57BL/6 mouse 10 weeks following hemispheric 30-Gy (50% isodose) GK-irradiation. Each panel shows twelve contiguous, 1-mm thick transaxial slices. Both the T1W and T2W images are indistinguishable from the corresponding images of non-irradiated mice and show no evidence of radiation-induced tissue damage.

**Figure 1A** shows representative contrast-enhanced, T1-weighted images of GL261 tumors growing in RI^2^M (top panels) and non-irradiated (bottom panels) mouse brain. Irradiated brains received 30 Gy (50% isodose) of GK radiation six weeks prior to tumor implantation. As demonstrated in **Figure 2**, which shows T2-weighted images and contrast-enhanced, T1-weighted images of mouse brain collected ten weeks post-GK-irradiation (30 Gy @ 50% isodose), no acute radiation effects or blood-barrier breakdown are observed at the time of tumor implantation (10, 14). Nonetheless, as is evident in the images in **Figure 1A**, tumors in previously irradiated brain grow more aggressively than corresponding tumors in non-irradiated mice as reflected in the increased size and invasiveness of the lesions. **Figure 1B** shows plots of mean lesion volume as a function of time post-tumor-implantation for cohorts of irradiated (n=9) and non-irradiated (n=10) mice. A trend in lesion volumes (irradiated *vs.* non-irradiated brain) is observed on post-implantation day 14, and the difference is statistically significant on day 18 (p=0.03).

### Tumors Growing in Previously Irradiated Mouse Brain Do Not Respond to Anti-PD-L1 Immunotherapy

**Figure 3:**
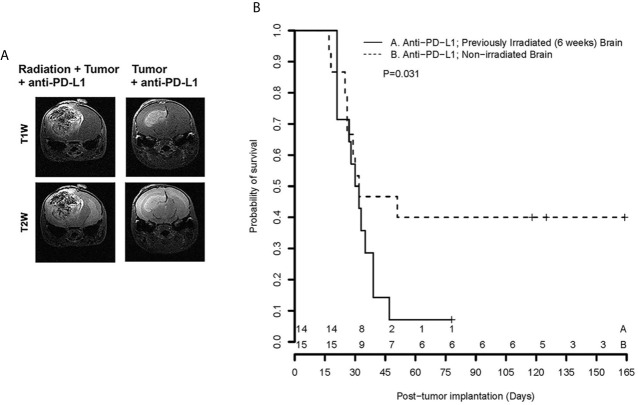
**(A)** Representative contrast-enhanced, T1- and T2-weighted MR images of the brains of C57Bl/6 mice, previously irradiated focally, or not, implanted with GL261 cells in the irradiated hemisphere and treated with anti-PD-L1 therapy; **(B)** Kaplan-Meier curves show that survival of tumor-bearing mice whose brains were not irradiated (n = 15; dashed line) is significantly greater than for mice whose brains were irradiated six weeks prior to tumor implantation (n = 14; solid line), p = 0.031 [extended log-rank test (22)]. + signs indicate mice that were sacrificed for histology; numerals near the bottom of the plot reflect the number of surviving mice in groups A (Anti-PD-L1; RI^2^M) and B (Anti-PD-L1; non-irradiated brain).

**Figure 3** (left) displays representative contrast-enhanced, T1- and T2-weighted MR images of tumors growing in mouse brain, collected on post-implantation day 21, for subjects treated with anti-PD-L1 mouse monoclonal antibody on post-implantation days 3, 6, 9, 12, and 15. Early therapeutic intervention prior to establishment of visible tumors was chosen to minimize the impact of the differences in growth kinetics observed at later time points. By post-implantation day 21, the therapeutic responsiveness to anti-PD-L1 inhibition of tumor in non-irradiated brain, compared with irradiated brain, is clearly evident. **Figure 3** (right) shows Kaplan-Meier survival curves for cohorts of tumor-bearing, anti-PD-L1-treated mice. The survival of tumor-bearing mice whose brains were not irradiated (n=15; dashed line) is significantly greater than for mice whose brains were irradiated six weeks prior to tumor implantation (n=14; solid line). The ~50% survival of anti-PD-L1-treated, non-irradiated mice is consistent with previously published studies of ICI-treated mice (21, 23–25).

### Activated Microglia Are More Prevalent in Irradiated Mouse Brain

**Figure 4:**
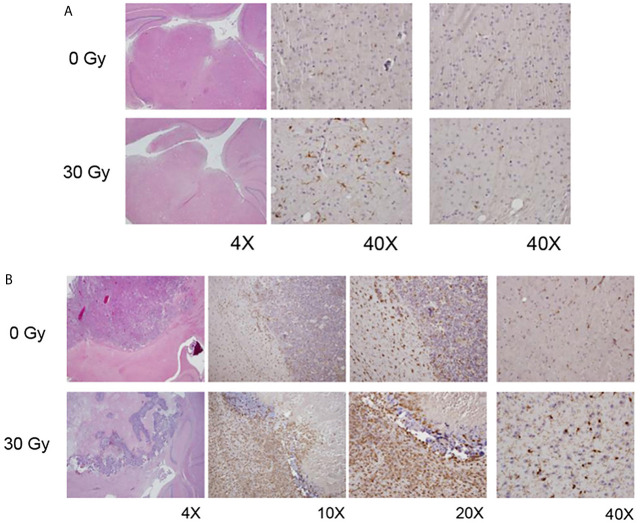
**(A)** H&E (left column) and IBA1 images (middle, right columns) from non-irradiated (0 Gy; top panel) and focally irradiated (30 Gy; bottom panel) mice in the absence of tumor. Increased IBA1 staining, indicative of increased levels of activated microglia and macrophages, is observed in the irradiated hemisphere, which looks normal by standard H&E staining. **(B)** H&E (left column) and IBA1 (all other columns) images from non-irradiated (0 Gy; top panel) and irradiated (30 Gy; bottom panel) tumor-bearing mice. Non-irradiated mice generally have well-circumscribed tumor with microglial activation both within the tumor and at its periphery. Tumors implanted into RI^2^M are characterized by large areas of hemorrhage and necrosis with lesser viable tumor. However, there was marked activation of microglia within the tumor and surrounding it.

Immunohistochemical staining utilized IBA-1 antibodies to identify activated microglia and macrophages. **Figure 4A** compares IBA-1 staining of non-irradiated and (focally) irradiated mouse brain, in the absence of tumor. Increased levels of activated microglia and macrophages are found in the irradiated hemisphere, which looks normal by standard H&E staining. Populations of activated microglia and macrophages on the non-irradiated side are comparable to those seen bi-hemispherically in non-irradiated mice.

**Figure 4B** displays IBA-1 staining of tumor-bearing mouse brain, with the top panel showing tumor growing in non-irradiated brain, and the bottom panel tumor growing in RI^2^M. Non-irradiated mice generally had solid well-circumscribed tumor with activated microglia/macrophages within the tumor and at the periphery. Rarely, cuffing of macrophages/microglial cells at the interface of tumor with normal parenchyma was also observed. When present, it was thin or partial and never as complete/circumferential and dense as seen in the irradiated groups. Outside of the tumor, there were substantial numbers of activated microglia and macrophages on the ipsilateral side, with little IBA-1 staining on the contralateral side. Tumors implanted into the RI^2^M were characterized by large areas of hemorrhage and necrosis with lesser viable tumor. However, a thick cuff of macrophages/microglial cells formed around the tumor and at its interface with the normal brain parenchyma. Interestingly, for larger tumors/lesions, the contralateral side also had increased numbers of activated microglia and macrophages compared to tumor in non-irradiated brain. Microglia/macrophages within the tumor itself were comparable in the two groups (data not shown).

**Figure 5:**
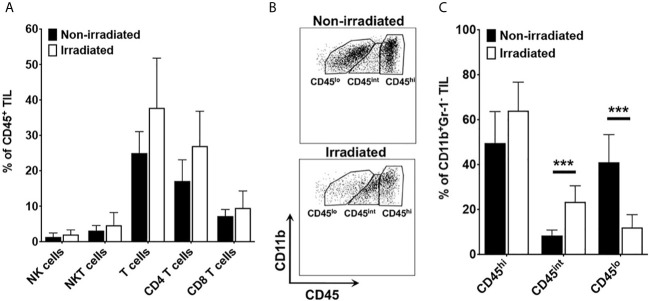
**(A)** quantifies the relative frequency of CD45^+^ tumor-infiltrating leukocytes (TIL) that are NK cells (CD3^-^NK1.1^+^), NKT (CD3^+^NK1.1^+^), total T cells (CD3^+^), CD8 T cells (CD3^+^CD8^+^), and CD4 T cells (CD3^+^CD4^+^). **(B)** is a representative flow plot demonstrating the gating strategy to identify resting microglia (CD45^lo^), activated microglia (CD45^int^), and macrophages/monocytes (CD45^hi^). Cells are gated on lineage negative (i.e., Non-T cells, Non-NK cells), CD11b-positive, Gr-1-negative TIL). **(C)** quantifies the percentage of CD11b^+^ Gr-1^-^ TIL that are CD45^hi^, CD45^int^, or CD45^lo^. Data are representative of two independent experiments with 4-5 mice in each experiment. Black bars represent non-irradiated mice; white bars previously irradiated mice. Differences in CD45^int^ and CD45^lo^ are statistically significant (***p < 0.0005).

Having observed a thick circumferential cuffing of activated macrophages/microglia surrounding tumor implanted in previously irradiated brain, we hypothesized that one mechanism of acquired resistance to anti-PD-L1 inhibition therapy in previously irradiated mice is exclusion of effector T cells from homing into the tumor microenvironment. Using fluorescence-activated cell sorting (FACS), changes in immune cell populations in orthotopic mouse tumors grown in RI^2^M *vs.* non-irradiated brain were compared. There were no differences in absolute number or relative frequency of T cell subsets or NK/NKT cells (**Figure 5A**). However, there were significant differences in the relative frequency of myeloid subpopulations in tumor-bearing mice. **Figure 5B** is a representative flow plot demonstrating the gating strategy used to identify resting microglia (CD45^lo^), activated microglia (CD45^int^), and macrophages/monocytes (CD45^hi^) within the CD11b^+^ Gr-1^-^ subpopulation. **Figure 5C** quantifies the relative frequency of CD11b^+^ Gr-1^-^ TIL that are CD45^hi^, CD45^int^, or CD45^lo^. Overall, the difference in total number of microglia (resting + activated) between irradiated and non-irradiated groups is not statistically significant (data not shown), suggesting that irradiation does not induce proliferation of microglia. However, there is a significant difference in the relative frequency of activated *vs.* resting microglia in the irradiated tumor-bearing mice. Thus, these results suggest that the impact of irradiation on the brain parenchyma is primarily in changing the activation state of microglia.

## Discussion

Radiation is a key component of early therapeutic strategies for many malignant brain tumors and improves overall survival in newly diagnosed glioblastoma. The late effects of irradiation on the tumor microenvironment have an important impact on response to subsequent systemic therapies for treatment of recurrent malignant tumors. A better understanding of the immune and vascular components of the brain microenvironment is needed for improving clinical treatment strategies in patients with recurrent tumors.

We have developed a novel GK-enabled focal brain-irradiation mouse model (10) that provides a powerful platform for investigation of the late (six weeks) post-irradiation induced immunologic modulation of the brain tissue microenvironment. We emphasize that ours is not a model of radiotherapy aimed at treating existing brain tumors, but instead was developed for studying the growth and response to immunotherapy of tumors growing in previously irradiated brain. Consistent with our recently published study of naïve (non-irradiated) DBT tumors growing in the RI^2^M of Balb/C mice (10), naïve GL261 cells orthotopically implanted in the RI^2^M of C57Bl/6 mice show similarly remarkable changes in growth characteristics (**Figure 1**). Specifically, such tumors display aggressive, invasive growth, characterized by viable tumor and large regions of hemorrhage and necrosis, compared to tumors growing in non-irradiated brain. In short, tumors originating from naïve (non-irradiated) tumor cells orthotopically implanted in previously irradiated brain show many of the histologic hallmarks of recurrent glioblastoma in patients, features that are not observed in tumor cells growing in non-irradiated brain parenchyma. The model has direct clinical relevance because recurrent GBM is almost always growing in previously irradiated brain. Prior irradiation dramatically affects the growth and histologic features of tumors in the orthotopic mouse model.

Previous radiation altered the brain microenvironment, resulting in dramatic loss of sensitivity to anti-PD-L1 treatment in our mouse model. We remind the reader that our studies were designed to explore the effects of previous brain irradiation on the efficacy of immunotherapy, as distinct from therapy studies on existing tumors employing concomitant radiation and checkpoint inhibition (18, 19). The survival data reported in **Figure 2** are consistent with radiation generating an increased immunosuppressive tumor microenvironment that promotes therapeutic resistance and loss of sensitivity to PD-L1 therapy in aggressive recurrent mouse gliomas. This finding has significant implications for the rational design of immunotherapy trials in brain tumors. Specifically, a better understanding of the late effects of radiation on the brain/tumor microenvironment will be crucial to identifying effective therapies that can safely synergize with immune checkpoint inhibitors to enhance immune response and improve outcome for brain tumor patients.

Given the differences in tumor growth kinetics between tumor cells implanted in irradiated and non-irradiated brains, we initiated PD-L1 inhibition therapy early (day 3) post-implantation to avoid confounding variables associated with differences in tumor size. Thus, to see differences in survival following PD-L1 inhibition therapy was somewhat surprising. We reasoned that the impact of prior irradiation on the microenvironment and its impact on responsiveness to PD-(L)1 inhibition therapy was present at the time of implantation of tumor cells, rather than after the tumor was established. Therefore, we initially evaluated the primary immune cell subset present within the brain parenchyma at steady state, the microglia. As demonstrated by IBA-1 staining in **Figure 4**, microglia/macrophages do not proliferate in response to irradiation but rather acquire an activated phenotype (i.e., IBA-1 positive) in post-irradiated brain parenchyma, even in the absence of tumor. The observation of increased activated microglia following radiation is aligned with the chronic activation of microglia reported following irradiation in adult rats (26) and in C57BL/6 mice (27). In previously irradiated, tumor-bearing mice, there was significant activation of microglia and macrophages in and around the tumor, forming a thick ring of macrophages/microglial cells around the tumor and at its interface with the normal brain parenchyma. These IHC observations were further corroborated with flow cytometry experiments. Results reported in **Figure 5** demonstrate that there is a statistically significant increase in the relative frequency of activated microglia and a decrease in resting microglia among TIL isolated from irradiated compared to non-irradiated tumor microenvironment. The FACS results are consistent with and provide complementary support to the conclusions drawn from IBA-1 IHC. The changes in the orthotopic tumors growing in previously irradiated brain are consistent with an increased immunosuppressive environment and subsequent loss of response to immune checkpoint blockade.

The tumor-permissive and immunosuppressive characteristics of tumor-associated macrophages (TAM) have driven interest in development of novel therapeutic strategies to target these cells. Colony stimulating factor (CSF-1) is an important cytokine involved in survival, proliferation, and differentiation of tissue macrophages and their precursors. As a consequence, there has been considerable interest in CSF1 and its receptor (CSF1R), and various approaches targeting either the ligand or the receptor are currently in clinical development. In addition to CSF-1/CSF-1R pathway inhibition, other myeloid-directed targets are also being developed. Kaneda and colleagues demonstrated that administration of a PI3K-gamma inhibitor resulted in improved responsiveness of the tumor (28). In addition to the CSF-1 pathway and PI3K-gamma, CD40 agonists (29) have also been shown to remodel the myeloid compartment and are being explored as microenvironment modulators to combine with ICI. Likewise, CD47 agonists are thought to have a similar effect on remodeling the myeloid compartment (30). Thus, identifying novel agents that target the microenvironment, namely the myeloid compartment, to sensitize tumors to PD-L1 inhibition therapy is an active area of investigation and may be particularly needed in the post-irradiation setting.

To summarize, in our GK-enabled hemispheric brain irradiation mouse model, the persistent (six weeks post-irradiation) effects of the irradiation on the brain microenvironment are shown to induce substantial changes in tumor growth characteristics and response to immunotherapy. Specifically, naïve (non-irradiated) GL261 tumors growing in the RI^2^M grew markedly more aggressively, with tumor cells admixed with regions of hemorrhage and necrosis, and showed a dramatic loss of response to anti-PD-L1 therapy compared to tumors in non-irradiated brain. IHC and FACS analyses demonstrated increased relative frequency of different myeloid cell infiltration and increased activated microglia, which correlated with the loss of sensitivity to checkpoint immunotherapy. We are currently performing experiments using high-dimensional single-cell techniques to define changes in myeloid cell populations. Metabolism studies can also contribute important insights towards understanding the enhanced tumor growth and lack of responsiveness to checkpoint inhibitors observed in our model. Ongoing studies in our lab are directed at developing Deuterium Metabolic Imaging (DMI) (31, 32) for characterization of the RI^2^M. While there are well-recognized imperfections in murine models *vs.* the human condition, the changes in tumor growth and loss of sensitivity to checkpoint inhibitors are not subtle and provide a framework that motivates further analysis of the late effects of the irradiated brain/tumor microenvironment on tumor growth and therapeutic efficacy.

## Data Availability Statement

The raw data supporting the conclusions of this article will be made available by the authors, without undue reservation.

## Ethics Statement

The animal study was reviewed and approved by Washington University Institutional Animal Care and Use Committee.

## Author Contributions

Conceptualization/Study Design: JG, KR, and JA. Data acquisition, analysis, and curation: XG, JE, TJ, LY, and SD. Statistical analysis: TJ and FG. Writing – original draft: JG and JA. Writing – review and editing: JG, TJ, CT, KR, and JA. All authors contributed to the article and approved the submitted version.

## Funding

This research was funded by the Alvin J. Siteman Cancer Center Investment Program (supported by the Foundation of Barnes-Jewish Hospital, Cancer Frontier Fund; National Cancer Institute, Cancer Center Support Grant, P30 CA091842; and Barnard Trust), NIH/NIBIB R01 EB029752, and Elekta Instrument AB (Stockholm, Sweden).

## Conflict of Interest

The authors declare that the research was conducted in the absence of any commercial or financial relationships that could be construed as a potential conflict of interest.
